# Hepatitis B Virus X Protein Modulates p90 Ribosomal S6 Kinase 2 by ERK to Promote Growth of Hepatoma Cells

**DOI:** 10.3390/v15051182

**Published:** 2023-05-17

**Authors:** Ning Han, Qingbo Zhang, Xiaoqiong Tang, Lang Bai, Libo Yan, Hong Tang

**Affiliations:** 1 Center of Infectious Diseases, West China Hospital of Sichuan University, Chengdu 610041, China; 2 Division of Infectious Diseases, State Key Laboratory of Biotherapy and Center of Infectious Disease, West China Hospital, Sichuan University, Chengdu 610041, China; 3 Jiangxi Qiushi Forensic Science Center, Nanchang 330096, China

**Keywords:** hepatitis B virus X protein, hepatocellular carcinoma, p90 ribosomal S6 kinase 2, cAMP response element binding protein, proliferation

## Abstract

Hepatitis B virus (HBV) infection is a leading cause of hepatocellular carcinoma (HCC), one of the most prevalent malignant tumors worldwide that poses a significant threat to human health. The multifunctional regulator known as Hepatitis B virus X-protein (HBx) interacts with host factors, modulating gene transcription and signaling pathways and contributing to hepatocellular carcinogenesis. The p90 ribosomal S6 kinase 2 (RSK2) is a member of the 90 kDa ribosomal S6 kinase family involved in various intracellular processes and cancer pathogenesis. At present, the role and mechanism of RSK2 in the development of HBx-induced HCC are not yet clear. In this study, we found that HBx upregulates the expression of RSK2 in HBV-HCC tissues, HepG2, and SMMC-7721 cells. We further observed that reducing the expression of RSK2 inhibited HCC cell proliferation. In HCC cell lines with stable HBx expression, RSK2 knockdown impaired the ability of HBx to promote cell proliferation. The extracellularly regulated protein kinases (ERK) 1/2 signaling pathway, rather than the p38 signaling pathway, mediated HBx-induced upregulation of RSK2 expression. Additionally, RSK2 and cyclic adenosine monophosphate (cAMP) response element binding protein (CREB) were highly expressed and positively correlated in HBV-HCC tissues and associated with tumor size. This study showed that HBx upregulates the expression of RSK2 and CREB by activating the ERK1/2 signaling pathway, promoting the proliferation of HCC cells. Furthermore, we identified RSK2 and CREB as potential prognostic markers for HCC patients.

## 1. Introduction

Hepatocellular carcinoma (HCC) is a prevalent malignant tumor worldwide that poses a significant threat to human health [1]. The patient’s underlying condition primarily limits the treatment strategy for HCC. For early-stage patients, surgery is the preferred treatment option and can provide the best prognosis. Radiofrequency ablation, transcatheter arterial chemoembolization, and stereotactic body radiation therapy also offer long-term survival opportunities for patients with poor underlying conditions [2]. However, 50% to 60% of patients still require systemic treatment with targeted therapies or immune checkpoint inhibitors [3]. With the continuous deepening of research on the molecular mechanisms and immune subtypes of HCC, new drugs targeting specific molecular mutations and novel immune checkpoint inhibitors are emerging one after another. In addition, methods based on tumor immunology theory, such as immunotherapy vaccines, adoptive T cell immunotherapy, and cytokine therapy, are gradually gaining a foothold [4]. Oncolytic virotherapy, which can specifically replicate and induce cancer cell apoptosis while preserving normal tissue from destruction, may also bring good treatment trends for resistant and orphan tumors [5]. With systemic treatment’s rapid development, combining multiple treatment methods brings new hope for HCC patients. Despite this, the treatment of HCC remains a major challenge.

One of the primary causes of HCC is hepatitis B virus (HBV) infection, which belongs to the *Hepadnaviridae* family—a group of small DNA viruses that exhibit host specificity and primarily target the liver [6]. The HBV genome comprises 3.2 kb of circular, partially double-stranded DNA with four open reading frames named pre-C/C, pre-S/S, P, and X [7]. The X gene encodes for the hepatitis B virus X protein (HBx). This multifunctional regulator interacts directly or indirectly with host factors to modulate gene transcription, signaling pathways, protein degradation, the cell cycle, cell proliferation, and apoptosis [8]. Any or all of these functions could potentially contribute to the development of HCC. While growing evidence shows that HBx plays a crucial role in promoting HCC, our understanding of the potential mechanisms underlying HBx regulation in HCC pathogenesis is still limited. In our earlier study, we utilized the iTRAQ proteomics technology to identify and quantify differentially expressed proteins in HepG2 cells with various functional domains of HBx and identified p90 ribosomal S6 kinase 2 (RSK2) as being highly expressed [9].

RSK2 belongs to the highly conserved serine/threonine kinase family, including four isoforms (RSK1, RSK2, RSK3, and RSK4), and is downstream of the mitogen-activated protein kinases (MAPKs) signaling pathway [10]. The importance of RSK2 is evident in its involvement in several vital biological processes, such as the regulation of gene transcription, cell cycle, proliferation, and differentiation, as well as cell survival and programmed cell death [11,12]. The identification of RSK2’s involvement in the development of various cancer types, including skin, prostate, colon, and breast cancer, has been revealed in recent studies, leading to increased attention in tumor research [13,14,15,16]. Research has demonstrated that RSK2 can be triggered by extracellular stimuli, including viruses, cytokines, growth factors, and environmental stress, leading to phosphorylation and activation of RSK2. Subsequently, downstream transcription and epigenetic factors are activated, resulting in cell proliferation and transformation into cancer cells and promoting cancer development [17]. RSK2 activates several substrates, such as cyclic adenosine monophosphate(cAMP) response element binding protein (CREB), activating transcription factor 1 (ATF1), activator protein 1, histone 13, and histone H2B, which play crucial roles in developing HCC. Therefore, it is hypothesized that RSK2 might be instrumental in the pathogenesis of HCC.

This study aimed to confirm the impact of HBx on RSK2 expression and provide supporting evidence for RSK2’s role in promoting HCC cell proliferation, particularly its effect on HBx-induced proliferation. Next, we attempted to elucidate the pathways involved in regulating RSK2 by HBx. Finally, we sought to demonstrate the expression of RSK2 and the downstream transcription factor CREB in cancer tissues associated with HBV-related HCC and evaluate their prognostic significance for patients.

## 2. Methods

### 2.1. Clinical Specimens

Specimens were collected from 92 patients at the West China Hospital of Sichuan University, including 76 HBV-HCC patients and 16 normal liver tissues, and all samples were frozen at −80 °C. Clinical data on the patients were obtained from the electronic medical records of West China Hospital, and prognosis information was obtained by telephone follow-up. The study was approved by the Ethics Committee of West China Hospital, Sichuan University, and the experiments were performed following the regulations of the Ethics Committee.

### 2.2. Cell Lines, Transfection, and Treatment

HepG2 cells and SMCC-7721 cells were cultured in high-glucose Dulbecco’s modified Eagle’s medium (Gibco, Grand Island, NY, USA) or Roswell Park Memorial Institute 1640 medium (Gibco, Grand Island, NY, USA) containing 10% FBS (Hyclone, Logan, UT, USA), penicillin (100 µg/mL), and streptomycin (100 µg/mL) at 37 °C and 5% CO_2_. HepG2 and SMCC-7721 cells that stably express HBx and knockdown RSK2 were prepared and selected in the presence of 400 µg/mL geneticin or 2 µg/mL puromycin for 2–3 weeks. Subsequently, cells stably down-regulating RSK2 expression were constructed based on HepG2-HBx and SMCC-7721 and selected with 2 µg/mL of puromycin.

### 2.3. Immunofluorescence

Cells were washed 3 times in pre-cooled phosphate buffered solution (PBS), fixed with 4% paraformaldehyde for 10 min, washed three times with PBS, incubated with 0.2% Triton X-100 for 15 min, washed three times with PBS, and blocked for 30 min. Anti-RSK2 (Cell Signaling Technology, Danvers, MA, USA, 5528) diluted 1:500 was incubated at room temperature for 1 h, washed three times with PBS, and a specific secondary antibody was incubated at room temperature in the dark for 1 h and washed three times with PBS. Nuclei were stained with 4′,6-diamidino-2-phenylindole (1 µg/mL; Roche, Switzerland) for 15 min at 37 °C and washed with PBS. Cells were mounted in a fluorescent mountant (Agilent, Santa Clara, CA, USA), and images were collected using a fluorescent microscope, counted with Image J software, and the number of RSK2-positive cells was calculated.

### 2.4. Cell Counting Kit-8(CCK-8) Proliferation Assay

Cells in the logarithmic growth phase were seeded in 96-well plates (100 µL/well). According to the grouping, four wells were set up for each group, and plasmid transfection was carried out. The culture plate was incubated at 37 °C and 5% CO_2_ for 24, 48, and 72 h, respectively. Afterward, 10 µL of CCK-8 solution (Sigma-Aldrich, Shanghai, China) was added to each well and incubated for 4 h. Using a microplate reader (Thermo Multiskan MK3, Thermo, MA, USA), the absorbance of each well was determined at 450 nm.

### 2.5. 5-Ethynyl-2′-deoxyuridine (EdU) Incorporation Assay

Incorporated EdU was detected with the Cell-Light EdU Apollo 567 (catalog no. C10310-1; RiboBio, Guangzhou, China), according to the manufacturer’s protocol. Cells in the logarithmic growth phase were seeded in 6-well plates at 1 × 10^6^ cells per well. Twenty-four hours after transfection, 1 mL of medium containing 50 µM EdU was added to each well based on the grouping and incubated for 2 h. The cells were then fixed with PBS containing 4% paraformaldehyde and decolorized with 2 mg/mL glycine. To permeabilize the cells, 100 µL of 0.5% TritonX-100 PBS was added to each well and incubated for 10 min on a shaker, followed by washing with PBS. Following that, 100 µL of 1× Apollo^®^ staining reaction solution was added to each well and incubated in the dark for 30 min. The wells were washed thrice on a decolorizing shaker with 100 µL of 0.5% TritonX-100 PBS, then 100 µL of 1× Hoechst 3324 reaction solution was added to each well. After incubating in the dark for 30 min, the wells were washed three times with PBS, and pictures were taken using a fluorescence microscope. The number of EdU-positive cells was then calculated by counting the cells using Image J software.

### 2.6. Real-Time Quantitative Polymerase Chain Reaction (RT-qPCR) Analysis

Total RNA was extracted using the Trizol reagent (Invitrogen, Carlsbad, CA, USA). One microgram of RNA was reverse-transcribed into cDNA with the PrimeScript™ RT reagent Kit with gDNA Eraser (Takara, Japan). Quantitative RT-qPCR was performed on the LightCycler 96 System (Roche, Germany) using the FastStart Essential DNA Green Master (Roche, Germany) according to the manufacturer’s instructions. The relative mRNA expressions of RSK2 were calculated with the 2^−ΔΔct^ method and normalized with GAPDH. The primers used were: RSK2 (F: 5′-CGTGGCAGAAGATGGCTGTG-3′, R: 5′-CTGCCTTTTCATGTCCTTCCT-3′); HBx (F: 5′-ACCGACCTTGAGGCCTACTT-3′, R: 5′-GCTTGGCAGAGGTGAAAAAG-3′); and GAPDH (F: 5′-AGAAGGCTGGGGCTCATTTG-3′, R: 5′-AGGGCCATCCACAGTCTTC-3′).

### 2.7. Western Blot

Cells or tissues were resuspended in radioimmunoprecipitation assay buffer and a protease inhibitor cocktail at a ratio of 100:1, lysed on ice for 30 min, and placed into a 1.5-mL eppendorf tube. Following that, the supernatant was obtained after centrifugation at 4 °C and 12,000× *g* for 15 min. Next, 5× sodium dodecyl sulfate (SDS) loading buffer was added to the supernatant at a ratio of 4:1 according to the amount of supernatant, and the samples were placed in an incubator at 95 °C for 5 min. Finally, the protein concentration was determined with the bicinchoninic acid assay method. The solution containing 40 µg of protein was used as the sample volume. Samples were separated by SDS-polyacrylamide gel electrophoresis at 70 V for 30 min and 110 V for 90 min. The proteins were transferred to a polyvinylidene difluoride membrane by electroporation using the rapid protein transfer system. After transfer to the membrane, the membranes containing a band for the target protein were incubated with 5% skim milk for 1 h and washed three times for 5 min each. The membranes were incubated with antibodies in a shaker at 4 °C overnight. After washing three times, the membranes were incubated with Tris buffered saline buffer with tween 20 (TBST) containing goat anti-rabbit IgG and anti-horseradish peroxidase while protected from light for 2 h. TBST was used to wash the membranes three times for 5 min each, and the membranes were finally scanned with the Bio-Rad ChemiPoc Imager. The antibodies involved are: RSK2 (Cell Signaling Technology, Danvers, MA, USA, 1:1000, 5528), p-RSK2 (Cell Signaling Technology, Danvers, MA, USA, 1:1000, 9601), CREB (Cell Signaling Technology, Danvers, MA, USA, 1:1000, 9197), Erk (Cell Signaling Technology, Danvers, MA, USA, 1:1000, 3552), p-Erk (Cell Signaling Technology, Danvers, MA, USA, 1:1000, 4370), p38 (Cell Signaling Technology, Danvers, MA, USA, 1:1000, 8690), and p-p38 (Cell Signaling Technology, Danvers, MA, USA, 1:1000, 4511). Anti-GAPDH (TA-08) was an internal reference purchased from Zhong Shan-Golden Bridge (Beijing, China).

### 2.8. Nude Mouse Tumorigenicity Experiment

Male mice (BALB/c, 6 weeks of age) (Huafukang Bio, Beijing, China) were injected subcutaneously with HCC cells (1 × 10^7^ cells per mouse). Tumor measurements were taken by caliper every 3 days. The tumor volumes were determined by measuring the length (l) and the width (w) and calculating the volume (V = lw^2^/2). After 18 days, the mice were killed, and tumors were excised, pictured, and weighed.

### 2.9. Immunohistochemistry (IHC)

The wax coating on the slices was removed by soaking them in water before treating them with 3% H_2_O_2_ at room temperature for 10 min. The slices were then rinsed with distilled water and washed in PBS twice for 5 min each time. Next, 5% normal goat serum was applied and incubated at room temperature for 10 min. After removing the serum, the cells were incubated with the appropriate antibody at 4 °C overnight. The slices were then washed with PBS three times and treated with horseradish peroxidase-labeled streptavidin at 37 °C for 30 min, followed by development with a chromogenic agent for 15 min. Finally, the sections were rinsed with water, stained again, dehydrated, cleared, and sealed.

### 2.10. Statistical Analysis 

Analysis was performed using SPSS 16.0 for Windows (SPSS Inc., Chicago, IL, USA). All quantitative data were recorded as mean ± S.D. The Student’s *t*-test, or Wilcoxon rank sum test, performed comparisons between two groups. Differences among multiple groups were assessed by one-way ANOVA analysis or the Wilcoxon rank sum test. The Pearson χ^2^ test, or Fisher exact test, was used to compare qualitative variables. Kaplan-Meier analysis was used to assess survival. Statistical significance was defined as *p* < 0.05.

## 3. Results

### 3.1. HBx Up-Regulates RSK2 in Hepatoma Cells

**Figure 1:**
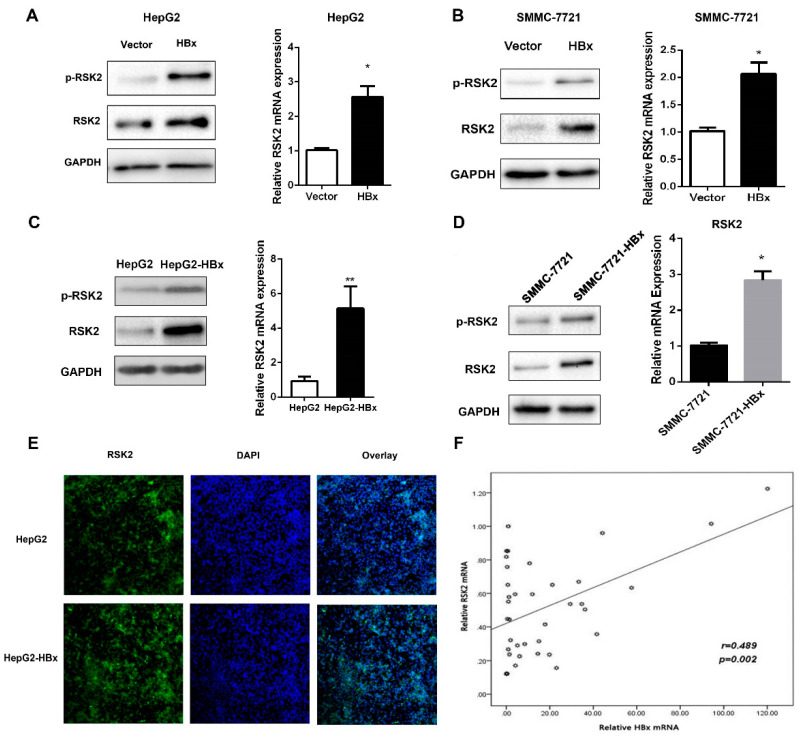
HBx upregulates the expression of RSK2 in HCC cells. (**A**) Relative mRNA and protein levels of RSK2 in HepG2 cells. Cells were transiently transfected with pNKF-HBx and pNKF-vector for 48 h. (**B**) Relative mRNA and protein levels of RSK2 in SMMC-7721 cells. Cells were transiently transfected with pNKF-HBx and pNKF-vector for 48 h. (**C**) RSK2 mRNA and protein levels in HepG2 cells stably expressing pNKF-vector and pNKF-HBx. (**D**) RSK2 mRNA and protein levels in SMMC-7721 cells stably express pNKF-vector and pNKF-HBx. (**E**) Fluorescence intensity of RSK2 in HepG2 cells stably expressing pNKF-vector and pNKF-HBx. Green and blue represent RSK2 positivity and nuclear positivity, respectively. (**F**) Correlation of HBx and RSK2 mRNA expression levels in 38 HBV-HCC patients’ cancer tissues (r = 0.489, *p* = 0.002). Student’s *t*-test was used to calculate *p* values, represented as * *p* < 0.05; ** *p* < 0.01.

To explore whether HBx can affect RSK2 expression in HCC cells. We transiently overexpressed HBx in HepG2 and SMMC-7721 cells, respectively, and detected the expression level of RSK2 using Western blot and RT-qPCR. Both mRNA and protein levels of RSK2 were higher in cells transiently transfected with pNKF-HBx compared to the control group (Figure 1A,B). A similar effect of RSK2 upregulation was observed in HepG2-HBx cells and SMMC-7721-HBx cells, which stable express HBx (Figure 1C,D). Compared with the control group, the fluorescence intensity and quantity of RSK2 in HepG2-HBx cells were stronger (Figure 1E). We also randomly extracted RNA from 38 HBV-HCC patients’ cancer tissues, detected the mRNA levels of HBx and RSK2 with RT-qPCR, and performed correlation analysis. We found a positive correlation between HBx mRNA and RSK2 mRNA (r = 0.489, *p* = 0.002, Figure 1F). These data suggest that the HBV viral protein HBx can upregulate RSK2 expression and may be associated with HCC.

### 3.2. RSK2 Is Required for the Proliferation of Hepatoma Cells

**Figure 2:**
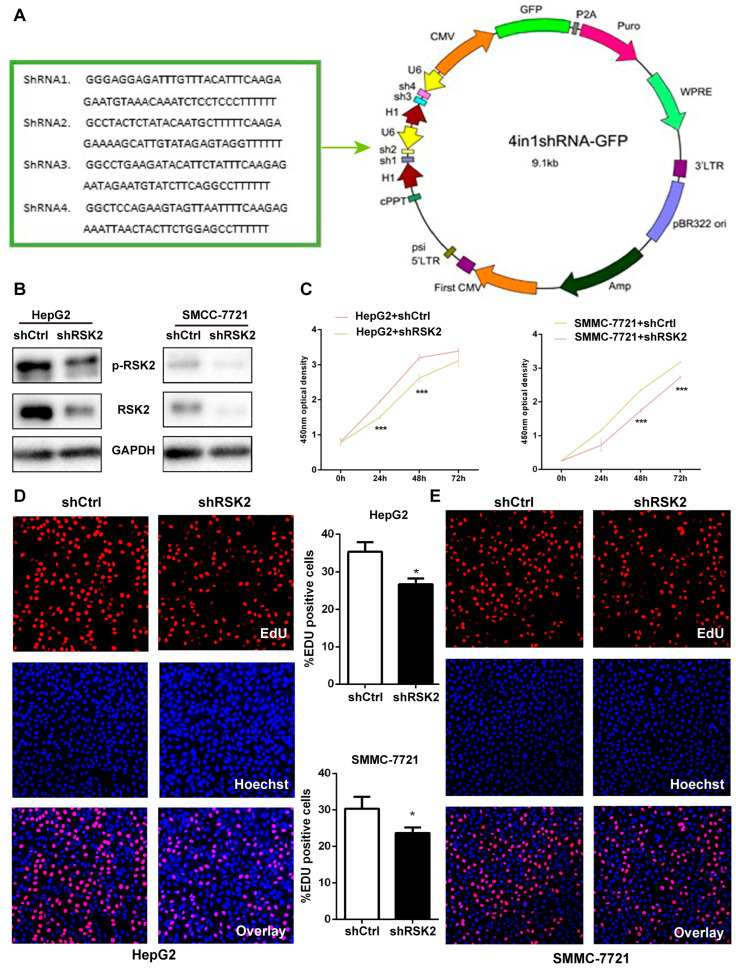
RSK2 promotes the proliferation of HCC cells. (**A**) Pattern diagram of the RSK2 shRNA plasmid. (**B**) Protein levels of RSK2 and p-RSK2 in HepG2 and SMMC-7721 cells after transfection with a control shRNA plasmid (shCtrl) and a RSK2 shRNA plasmid (shRSK2). (**C**) The 450 nm optical density (OD) values of HepG2 and SMMC-7721 cells transfected with a control shRNA plasmid (shCtrl) and a RSK2 shRNA plasmid (shRSK2) for 24 h, 48 h, and 72 h. (**D**) HepG2 cells were transfected with a control shRNA plasmid (shCtrl) and a RSK2 shRNA plasmid (shRSK2) for EdU staining, and the average percentage of EdU-positive cells in HepG2 cells decreased from 35.3% to 26.7%. Red is the RSK2-positive signal, and blue is the positive nuclear signal. (**E**) SMMC-7721 cells were transfected with a control shRNA plasmid (shCtrl) and a RSK2 shRNA plasmid (shRSK2) for EdU staining, and the average percentage of EdU-positive cells decreased from 30.3% to 20.7%. Red is the RSK2-positive signal, and blue is the positive nuclear signal. Student’s *t*-test was used to calculate *p* values, represented as * *p* < 0.05, *** *p* < 0.001.

To clarify the role of RSK2 in hepatocarcinogenesis, we downregulated the expression of RSK2 in HCC cell lines to observe its effect on proliferation. Initially, RSK2 shRNAs 1-4 were designed based on the gene sequence, and the four target sequences were cloned into the 4in1shRNA-GFP vector to knockdown the expression of RSK2 (Figure 2A). In HepG2 and SMMC-7721 cells, the shContrl and shRSK2 plasmids were transferred, respectively, and the Western blot found that the levels of RSK2 and p-RSK2 decreased, suggesting that the shRSK2 plasmid was effective (Figure 2B). To explore whether down-regulated RSK2 expression promotes HCC proliferation, we performed the CCK8 cell proliferation assay and the EdU staining assay, respectively. In the RSK2 knockdown of HepG2 and SMMC-7721 cells, the 450 nm optical density (OD) values were decreased, suggesting that reducing RSK2 expression could inhibit the proliferation of HCC cells (Figure 2C). As shown in Figure 2D,E, the mean values of the proportion of EdU-positive cells in HepG2 cells and SMMC-7721 cells decreased from 35.3% and 30.3% to 26.7% and 20.7%, respectively, after the knockdown of RSK2 expression. The number of EdU-positive cells in HepG2 and SMMC-7721 cells decreased after inhibition of RSK2 expression, suggesting that RSK2 may be involved in cell proliferation.

### 3.3. RSK2 Is Required for HBx-Induced Proliferation of Hepatoma Cells

**Figure 3:**
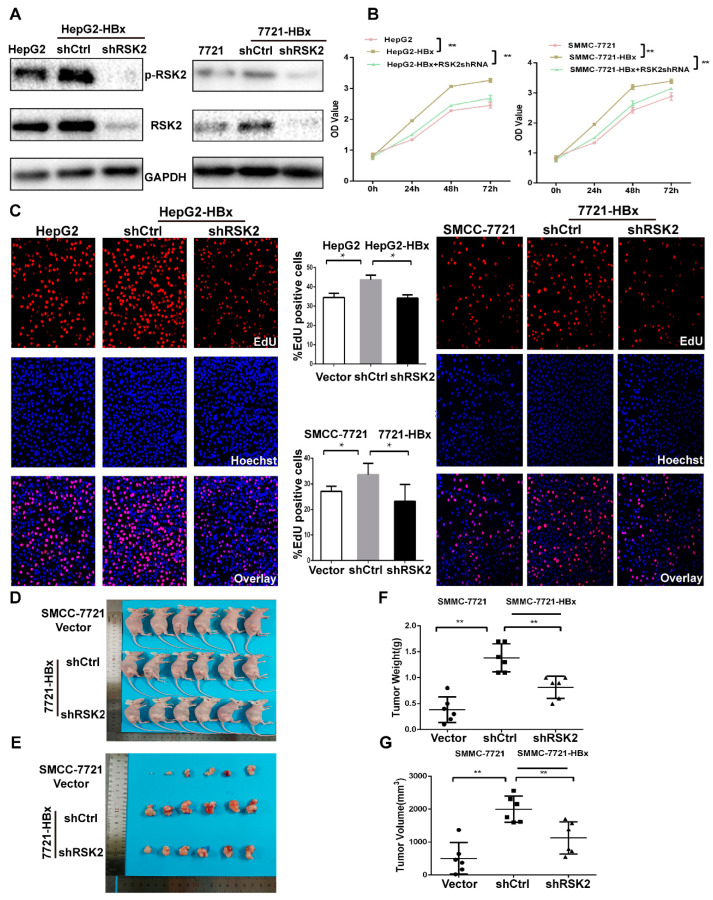
Reduction of RSK2 expression inhibits HBx-induced HCC cell proliferation. (**A**) The RSK2 shRNA plasmid and control plasmid were transfected in HepG2-HBx cells and SMMC-7721-HBx cells, and the protein levels of p-RSK2 and RSK2 were detected by Western blotting. (**B**) The 450 nm optical density (OD) values of HepG2 cells and SMMC-7721 cells for 24 h, 48 h, and 72 h. (**C**) EdU staining of HepG2 cells and SMMC-7721 cells. Red is the RSK2-positive signal, and blue is the positive nuclear signal. (**D**,**E**) In vivo mouse xenograft model and tumors derived from SMMC-7721 cells. (**F**,**G**) Statistical plots of weight and volume of tumors stripped from mouse xenograft models. Student’s *t*-test was used to calculate *p* values, represented as * *p* < 0.05, ** *p* < 0.01.

To elucidate whether HBx promotes HCC cell proliferation through upregulation of RSK2 expression, cells with stable knockdown of RSK2 expression were successfully constructed in HepG2-HBx and SMMC-7721-HBx cells stably expressing HBx (Figure 3A). The results of the CCK8 cell proliferation assay suggested that stable expression of HBx promoted cell proliferation in both HepG2 cells and SMMC-7721 cells, while the ability of HBx to promote HCC cell proliferation was weakened after knockdown of RSK2 (Figure 3B). EdU proliferation assays also indicated that the proportion of EdU-positive cells was higher in HepG2-HBx cells than in HepG2 cells without HBx expression; knocking down RSK2 expression in HepG2-HBx cells decreased the proportion of EdU-positive cells, and a similar phenomenon was seen in SMMC-7721 cells (Figure 3C). Following that, the SMMC-7721 cells, SMMC-7721-HBx-shCtrl cells, and SMMC-7721-HBx-shRSK2 cells were inoculated in the skin of the medial thigh of nude mice (Figure 3D,E). The tumor volume and weight were measured 6 weeks after inoculation. The tumor weights were 0.38 g, 1.38 g, and 0.82 g, and the tumor volumes were 507.70 mm^3^, 2003.20 mm^3^, and 1125.00 mm^3^, respectively (Figure 3F,G). In general, through in vivo and in vitro experiments, we demonstrated that HBx could promote the proliferation of HCC cells, and reducing the expression of RSK2 can impair HBx-induced HCC proliferation.

### 3.4. HBx Regulates RSK2 Expression throughERK1/2 and Further Affects the Expression of Downstream Transcription Factor CREB

**Figure 4:**
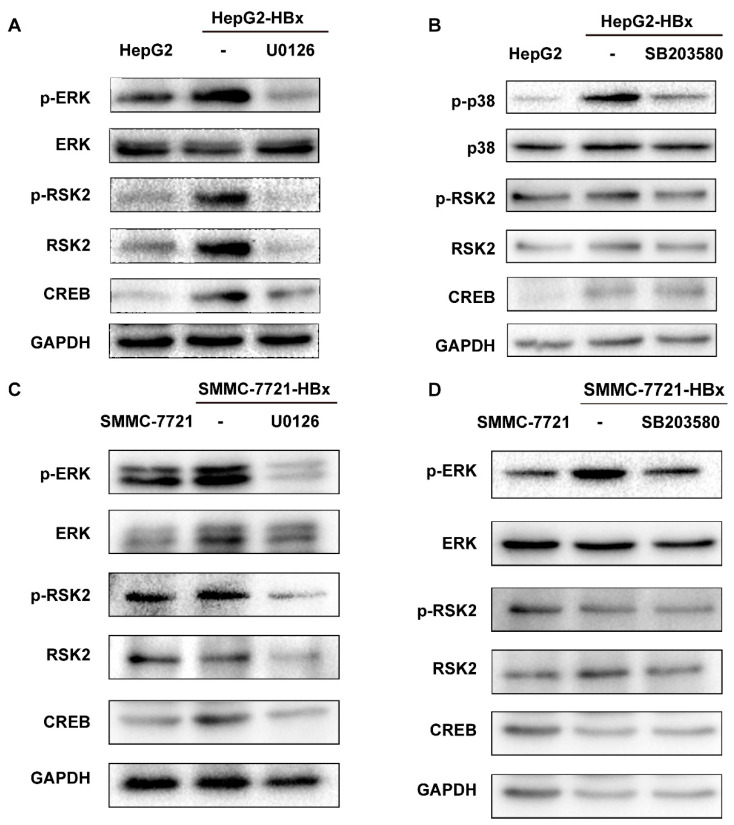
HBx regulates CREB expression through the ERK1/2/RSK2 axis. (**A**) Protein levels of ERK, RSK2, and CREB after treatment of HepG2-HBx cells with the ERK1/2 inhibitor U0126. (**B**) Protein levels of p38, RSK2, and CREB after treatment of HepG2-HBx cells with the p38 inhibitor SB203580. (**C**) Protein levels of ERK, RSK2, and CREB after treatment of SMMC-7721-HBx cells with the ERK1/2 inhibitor U0126. (**D**) Protein levels of p38, RSK2, and CREB after treatment of SMMC-7721-HBx cells with the p38 inhibitor SB203580.

RSK2 belongs to the highly conserved serine/threonine kinase family, is located downstream of ERK1/2 or p38, and is an important member of the MAPK signaling pathway. HepG2 and SMMC-7721 cells expressing HBx were treated with ERK1/2 inhibitor U0126 or p38 inhibitor SB203580, respectively, and both phosphorylated ERK and phosphorylated p38 were decreased, suggesting successful inhibition of the downstream pathways of ERK1/2 and p38 (Figure 4A–D). The effect of HBx to increase RSK2 expression was diminished after U0126 inhibition of ERK1/2, while SB203580 inhibition of p38 did not affect this (Figure 4A–D). Previous studies have shown that RSK2 regulates the expression of the downstream transcription factor CREB, and we simultaneously observed the protein levels of CREB and found that inhibition of ERK1/2 by U0126 resulted in the same decreased trend of RSK2 and CREB, while similar results could not be observed for the p38 inhibitor SB203580 (Figure 4A–D). Taken together, the results suggest that HBx may regulate the expression of the downstream transcription factor CREB through the ERK1/2/RSK2 axis. 

### 3.5. RSK2 and CREB Were Highly Expressed in HBV-Associated Liver Cancer Tissues

**Figure 5:**
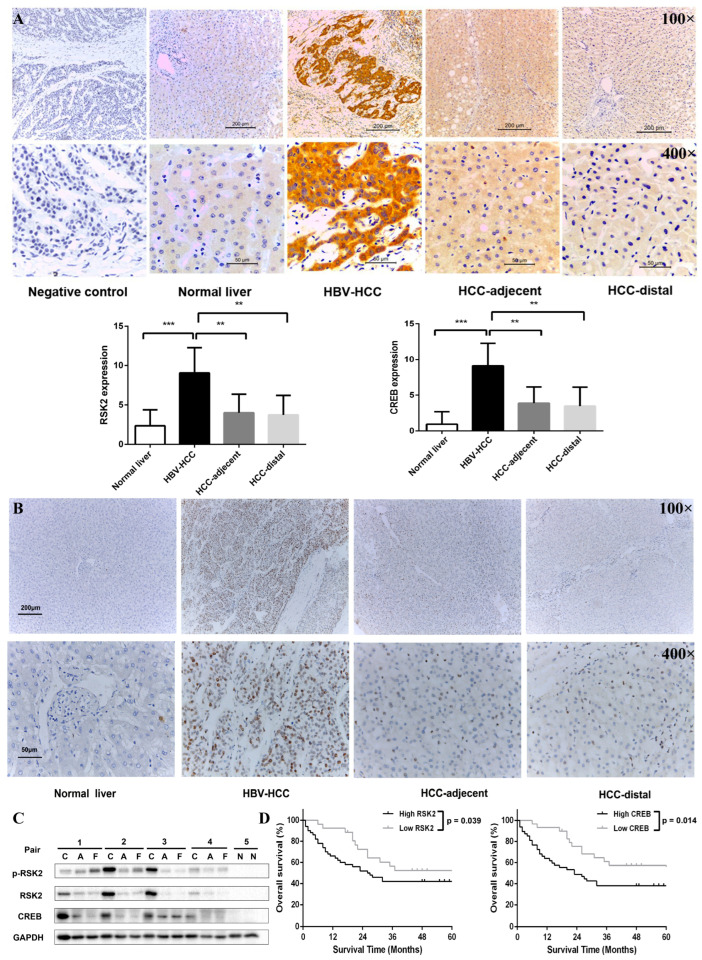
Expressions of RSK2 and CREB in HBV-HCC tissues and their relationship with clinical data. (**A**) Expression of RSK2 in patients with HBV-HCC and normal liver tissue. (**B**) Expression of CREB in liver tissue of patients with HBV-HCC and normal liver tissue. (**C**) Protein levels of RSK2 and CREB were detected by Western blotting in HBV-HCC cancer tissues (**C**), HBV-HCC-adjacent tissues (**A**), HBV-HCC-distal tissues (F), and normal liver tissues (N). (**D**) Postoperative Survival Analysis of HBV-HCC Patients. Student’s *t*-test was used to calculate *p* values, represented as ** *p* < 0.01, *** *p* < 0.001.

We further performed IHC staining of 76 HBV-HCC cancer tissues and 16 normal liver tissues. RSK2 expression was low in normal liver tissues and highest in HBV-HCC cancer tissues, with intermediate expression in HBV-HCC-adjacent and HBV-HCC-distal tissues (Figure 5A). 

CREB staining was performed on the same tissue microarrays, and CREB expression was significantly higher in HBV-HCC cancer tissues than in HBV-HCC-adjacent tissues, HBV-HCC-distal tissues, and normal liver tissues, with statistically significant differences (*p* < 0.001) (Figure 5B). Subsequently, the liver tissues of 4 HBV-HCC patients and 2 normal liver tissues were randomly selected for Western blot detection, and the results indicated that the expression of RSK2, p-RSK2, and CREB in HBV-HCC cancer tissues was significantly higher than in HBV-HCC-adjacent tissues, HBV-HCC-distal tissues, and normal liver tissues (Figure 5C). These results suggest that increased expression of RSK2 and CREB may play an important role in the pathogenesis of HBV-HCC.

Subsequently, we collected the clinical data of these 76 HBV-HCC patients and grouped them according to the IHC staining results of RSK2 and CREB, respectively. There were 63 (82.89%) people with high expression of RSK2 and 57 (75.00%) people with increased expression of CREB. Table 1 indicated that the number of patients with tumor diameters ≥5 cm was higher in the RSK2 and CREB high expression groups, suggesting that both RSK2 and CREB are associated with the growth of hepatocellular carcinoma. The average postoperative survival time of patients in the RSK2 high expression group was 36.1 months, compared to 50.1 months in the low-medium expression group, with a statistically significant difference (*p* < 0.05), suggesting the prognosis of HCC patients with high RSK2 expression was worse. Postoperative survival analysis of patients also indicated that increased expression of RSK2 and CREB may be associated with a worse prognosis (Figure 5D). 

**Figure 6:**
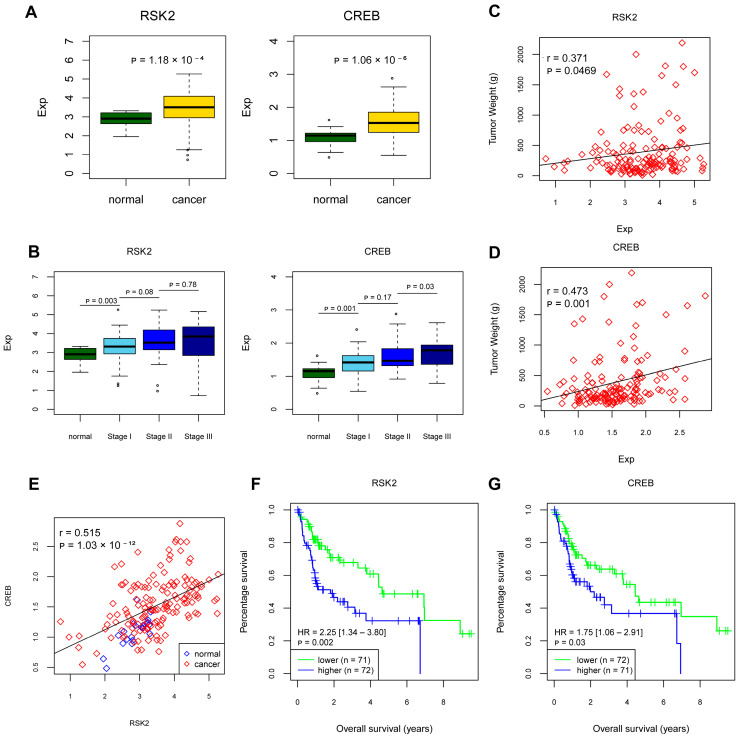
Correlation between the expression of RSK2 and CREB in the TCGA database and the clinical phenotype of HBV-HCC patients. (**A**) Differential expression of RSK2 and CREB in normal liver tissues and HBV-HCC liver cancer tissues. (**B**) Differential expression of RSK2 and CREB in HBV-HCC cancer tissues at different clinical stages. (**C**) Correlation analysis of RSK2 expression and tumor tissue weight. (**D**) Correlation analysis of CREB expression and tumor tissue weight. (**E**) Correlation analysis of RSK2 and CREB expression. (**F**,**G**) Survival curves of HBV-HCC patients grouped according to RSK2 expression and CREB expression, respectively. Student’s *t*-test was used to calculate *p* values, represented as * *p* < 0.05.

Considering the sample limitation, we downloaded the liver hepatocellular carcinoma data from the Cancer Genome Atlas (TCGA) database, selected HBV-HCC patients based on the serological results, and finally obtained transcriptome data and clinical data of 143 HBV-positive related HCC patients. The expressions of RSK2 and CREB in HBV-HCC patients were higher than those in normal patients (Figure 6A). In patients with HBV-HCC liver cancer, the expression levels of RSK2 and CREB were also related to the clinical stage (Figure 6B). Pearson correlation analysis of RSK2 and CREB expression with tumor weight showed that RSK2 was correlated with tumor size (r = 0.371, *p* = 0.0469, Figure 6C) and CREB was associated with tumor size (r = 0.473, *p* = 0.001, Figure 6D). A positive correlation between RSK2 and CREB expression was also found (r = 0.515, *p* < 0.001, Figure 6E). Kaplan-Meier survival analysis showed that the overall survival time of patients with high RSK2 expression was shorter than that of those with low RSK2 expression (*p* = 0.002), as well as CREB expression (*p* = 0.03) (Figure 6F,G). Taken together, RSK2 and CREB expression may be positively associated with HBV-HCC and may serve as a prognostic marker. 

## 4. Discussion

Globally, hepatocellular carcinoma (HCC) is the main type of liver cancer, accounting for about 75% of the cases [18]. Chronic HBV infection is the leading cause of HCC, and the economic burden is increasing [19,20]. Due to its large population, China has the highest number of HCC cases, and the years of life lost due to HCC account for nearly half of the global burden of liver cancer [21]. In China, HCC accounts for 93.0% of all primary liver cancers, of which 84.4% of HCC patients are positive for hepatitis B surface antigen and hepatitis B core antibody. HBV-related HCC has an earlier onset and a worse prognosis than HCV-related HCC [22]. Among the HBV genomes, HBx is believed to be the most closely related to chronic liver inflammation and HCC, attracting widespread attention. HBx is a co-transcriptional activator that interacts with multiple factors related to transcription and gene regulation in the cell through its activation domain. It widely activates viral and host factor promoters and regulates various signaling pathways in the cell, including those related to cell cycle, proliferation, apoptosis, DNA repair, and other factors closely associated with developing HCC [23]. Clarifying the host factors that play a key role in promoting the event and development of HCC through HBx and exploring the specific molecular mechanisms by which HBx regulates key factors may provide effective therapeutic targets for HBV-related HCC treatment.

In our study, we observed an upregulation of both mRNA and protein levels of RSK2 in HepG2 and SMMC-7721 cell lines, both in transient and stable expression of HBx. Moreover, a positive correlation was noted between the expression of HBx and RSK2 in HCC tissues. It has been shown that Kaposi’s sarcoma-associated herpesvirus ORF45 also significantly activates the expression of RSK2 [24]. According to several studies, RSK2 is closely associated with the development of various tumors, and its expression is significantly higher in tumor tissues such as glioblastoma, osteosarcoma, skin cancer, and squamous cell carcinoma compared to normal tissues [25,26,27,28]. RSK2 has been found to promote neoplastic cell transformation, possibly by phosphorylating and activating the Ser63 site of ATF1, thus regulating cell cycle progression and proliferation [29]. In order to clarify the involvement of RSK2 in hepatocarcinogenesis, we conducted knockdown experiments of RSK2 expression in HepG2 and SMMC-7721 cells, which inhibited proliferation. Further experiments, both in vivo and in vitro, involving cell lines stably expressing HBx revealed that HBx enhances the proliferation and growth of HCC cells. Notably, reducing RSK2 expression was found to impair HBx’s ability to promote the proliferation and development of HCC cells. 

RSK2, a highly conserved serine/threonine kinase family member, is downstream of the Ras/Raf/MAPK signaling pathway. Although no prior research has explored the regulatory role of HBx on RSK2, previous studies have suggested that HBx activates the Ras/Raf/MAPK signaling pathway [30,31,32]. Therefore, we investigated whether HBx regulates the expression of RSK2 by activating the MAPK signaling pathway. According to our findings, using U0126, an inhibitor of ERK1/2, can successfully reduce the levels of p-ERK1/2 in both HepG2 and SMMC-7721 cells. As a result, HBx loses its ability to upregulate the expression of RSK2, whereas the p38 inhibitor SB203580 does not significantly affect the upregulation of RSK2 induced by HBx. CREB is a vital downstream transcription factor in the MAPK signaling pathway. Our findings show a similar trend to RSK2, where inhibition of ERK1/2 led to ineffective upregulation of CREB expression by HBx. A recent study showed that inactivating mutations of RSK2 may occur in HCC patients, reducing the negative feedback inhibition of Son of sevenless 1/2 on the Ras/Raf/MAPK signaling pathway and ultimately leading to the proliferation and migration of Hep3B cells. In addition, the study also showed that RSK2 might also affect the synthesis of cholesterol and thus affect the occurrence of liver cancer caused by non-viral factors [33]. Taken together, this study and our results both suggest that RSK2 affects the proliferation of HCC through the Ras/Raf/MAPK signaling pathway. Previous studies have found that 3-hydroxy-3-methylglutaryl-coenzyme A reductase inhibitors (statins) can inhibit the activation of ERK1/2 and limit cholesterol synthesis in HCC cells, ultimately inhibiting the proliferation of HCC cells and inducing apoptosis, suggesting that statins may have the potential for the treatment and prevention of HCC [34,35]. A recent meta-analysis also suggested that using statins can reduce the incidence of HCC in a dose-dependent manner [36]. Therefore, inhibitors targeting this signaling pathway may be an adjunctive therapy for HCC.

In our patient tissue samples with HBV-HCC, immunohistochemical staining revealed significantly higher RSK2 and CREB levels in cancer tissues compared to adjacent, distant, and normal liver tissues, consistent with the expression trend observed in other tumor tissues such as osteosarcoma, skin cancer, and prostate cancer [25,27,37]. It was suggested that RSK2 and CREB may play an important role in the occurrence and development of HBV-HCC. Analysis of the clinicopathological data of 76 HBV-HCC cases revealed that the medium-to-high expression rates of RSK2 and CREB were significantly higher in the tumor diameter ≥5 cm group than in the tumor diameter <5 cm group, suggesting that RSK2 and CREB expression may be associated with the development of tumors. In addition, patients with high RSK2 and CREB expression had shorter postoperative survival times and worse prognoses, suggesting that RSK2 and CREB may be used as adjuncts to determine the prognosis of HBV-HCC patients. Analysis of data from HBV-HCC patients derived from TCGA also indicated that the expression of RSK2 and CREB was significantly higher in tumor tissues than in normal tissues, was positively correlated with tumor size, and that patients with increased expression of RSK2 and CREB had a shorter survival time and a worse prognosis. Taken together, both RSK2 and CREB expression were increased in HBV-HCC cancer tissues, correlated with tumor size, and may also serve as prognostic markers for HBV-HCC patients.

However, the present study also has some limitations. First, in our research, HBx was found to activate the expression of RSK2 and CREB, but the exact mechanism has not been investigated in depth. Secondly, this study only involved two hepatocellular carcinoma cell lines, and it is also worth exploring whether the results of this study are suitable for more hepatocellular carcinoma cells, such as HBV-infected cells. Thirdly, due to the difficulty in obtaining samples, this study did not detect the expression of RSK2 and CREB in the liver tissue of CHB patients who did not develop HCC, and we will further improve it in the future. Finally, some pan-RSK inhibitors have been developed, such as LJH685, ILJ308, and BI-D1870 [38,39]. RSK2 inhibitors were not selected for experiments in this study because they are not RSK2-specific inhibitors. It was found that LJH685 inhibited the effects of HCC-1806 xenografts in vivo without adverse side effects on mouse body weight [40]. Therefore, combining stable RSK inhibitors with other medicines may become an effective clinical treatment strategy for HCC. To address the above issues, we will investigate them in future studies and report any further available results when they are updated.

In conclusion, HBx is involved in hepatocarcinogenesis by upregulating the expression of RSK2 and CREB through the ERK1/2 signaling pathway and promoting the proliferation of HCC. In addition, RSK2 and CREB are highly expressed in HBV-HCC tumor tissues, which can be used as an auxiliary indicator to judge the prognosis of patients.

## Figures and Tables

**Table 1:** Clinical characteristics and expression of RSK2 and CREB in patients with HBV-related HCC.

	RSK2 Expression			CREB Expression		
Variable	High (n = 63, 82.89%)	Low (n = 13, 17.11%)	*p*	High (n = 57, 75.00%)	Low (n = 19, 25.00%)	*p*
Age <55 ≥55	37 26	10 3	0.22	37 20	10 9	0.34
Sex Male Female	56 7	10 3	0.59	51 6	15 4	0.24
Differentiation Well/Moderate Poor	42 21	10 3	0.86	42 15	10 9	0.09
TNM stages Stage I/II Stage III/IV	42 21	9 4	0.47	39 18	12 7	0.67
Tumor diameter <5 cm ≥5 cm	19 44	8 5	0.03	14 43	13 6	0.001
Lymph node metastasis No Yes	59 4	10 3	0.35	52 5	17 2	0.82
Vascular tumor thrombus No Yes	39 24	8 5	0.98	36 21	11 8	0.68

## Data Availability

Data not published in the manuscript are available from the corresponding author.
